# Cell-free supernatant of probiotic bacteria exerted antibiofilm and antibacterial activities against *Pseudomonas aeruginosa*: A novel biotic therapy

**DOI:** 10.3389/fphar.2023.1152588

**Published:** 2023-06-15

**Authors:** Mariana Martins Drumond, Ana Paula Tapia-Costa, Elisabeth Neumann, Álvaro Cantini Nunes, Jorge Wanderson Barbosa, Diego E. Kassuha, Pamela Mancha-Agresti

**Affiliations:** ^1^ Centro Federal de Educação Tecnológica de Minas Gerais (CEFET/MG), Departamento de Ciências Biológicas, Belo Horizonte, Minas Gerais, Brazil; ^2^ Centro Federal de Educação Tecnológica de Minas Gerais (CEFET/MG), Programa de Pós Graduação em Engenharia de Materiais, Belo Horizonte, Minas Gerais, Brazil; ^3^ Instituto de Investigaciones en Ciencias Químicas, Facultad de Ciencias Químicas y Tecnológicas, Universidad Católica de Cuyo, San Juan, Argentina; ^4^ Departamento de Microbiologia, Instituto de Ciências Biológicas, Universidade Federal de Minas Gerais, Belo Horizonte, Minas Gerais, Brazil; ^5^ Laboratório de Genética Molecular de Protozoários Parasitas, Departamento de Genética, Instituto de Ciências Biológicas, Universidade Federal de Minas Gerais (UFMG), Belo Horizonte, Minas Gerais, Brazil; ^6^ Centro Federal de Educação Tecnológica de Minas Gerais (CEFET/MG), Departamento de Engenharia de Materiais, Belo Horizonte, Minas Gerais, Brazil

**Keywords:** biofilm, lactic acid bacteria, postbiotics fraction (supernatant), skin pathogens, *Pseudomonas aeruginosa*

## Abstract

**Aim:** This study aims to verify the antibacterial and antibiofilm action of cell-free spent medium (CFSM) from four lactic acid bacteria with potential probiotic characteristics (*Lactiplantibacillus plantarum*, *Lactobacillus acidophilus*, *Lactobacillus johnsonii*, and *Lactobacillus delbrueckii*) against two *Pseudomonas aeruginosa* strains.

**Main methods:** The minimum inhibitory concentration (MIC) and minimum bactericidal concentration (MBC) of the CFSM, antibacterial activity by analysing the formation of inhibition zones, and inhibition of planktonic cultures were determined. Whether an increase in the concentration of CFSM influenced the growth of pathogenic strains and the anti-adhesive activity of the CFSM in biofilm formation (crystal violet and MTT assays) were determined, which were all corroborated by using scanning electron microscopy.

**Key findings:** The relationship between the MIC and MBC values showed a bactericidal or bacteriostatic effect for all the cell-free spent media (CFSMs) tested for *P. aeruginosa* 9027™ and 27853™ strains. The CFSM supplemental doses of 18 or 22%, 20 or 22%, 46 or 48%, and 50 or 54% of *L. acidophilus*, *L. delbrueckii*, *L. plantarum*, and *L. johnsonii*, respectively, could completely inhibit the growth of both pathogen strains. The antibiofilm activity of the CFSM in three biofilm conditions (pre-coated, co-incubated, and preformed) demonstrated values ranging between 40% and 80% for biofilm inhibition, and similar results were observed for cell viability.

**Significance:** This work provides strong evidence that the postbiotic derived from different Lactobacilli could be practical as an adjuvant therapy for reducing the use of antibiotics, being a good candidate to overcome the growing challenge of hospital infections due to this pathogen.

## 1 Introduction

The gram-negative opportunistic human pathogen *Pseudomonas aeruginosa* is responsible for much of the morbidity and mortality in nosocomial settings (Serra et al., 2015), leading to a wide variety of life-threatening acute and chronic infections that compromise the soft tissues, bloodstream, lungs, and urinary tract (Driscoll et al., 2007), specifically in cancer, cystic fibrosis, and immunocompromised patients (Wu and Zhu, 2021). The mortality rates are over 35% for hospital-acquired *P. aeruginosa* infectious, like ventilator-associated pneumonia or bacteraemia (Lynch et al., 2017), and approximately 75% for sepsis in patients who have more than 40% of their total body surface burned (Atiyeh et al., 2005), the skin and soft tissues being the principal areas infected by *P. aeruginosa*, which represents 21% of all cases of bacteraemia (Wahab and Rahman, 2013). The colonisation of burned tissues by *P. aeruginosa* characteristically results in a significantly greater area of infection and thereby postpones or prevents the healing process (Gjødsbøl et al., 2006). Thus, this pathogen causes the highest mortality rate among gram-negative bacteria, proteases, and lipases—known as virulence factors—which are used to degrade host barriers and evade immune mechanisms, allowing bacterial invasion of the bloodstream from the skin and consequently leading to soft tissue infections (Wu et al., 2011).

*Pseudomonas aeruginosa* is a biofilm-forming bacterium with a high intercellular signalling pathway, the quorum sensing (QS) system; these processes represent bacterial social behaviours. The QS-controlled functions are critical for acute virulence since these processes appear to play an essential role in the development of biofilms (Passador et al., 1993; Parsek and Greenberg, 2005; Atkinson, 2007). Biofilms are considered an aggregation of microorganism communities, attached and growing either on biotic or abiotic surfaces, generating a protection matrix by secreting biochemical substances and forming a physical semipermeable barrier that limits the diffusion of molecules (Hall-Stoodley et al., 2004; Mihai et al., 2018). When compared to planktonic cells, the pathogen microorganisms present in biofilms are highly resistant to antimicrobial agents (Penesyan et al., 2015), and the development of antibiotic resistance by *P. aeruginosa* has been widely documented. Thus, there is an urgent need to look for alternative strategies to treat chronic, biofilm-mediated infections of *P. aeruginosa*.

Live probiotics are microorganisms that offer health benefits to the host by direct or indirect interactions between cells or through their released metabolites. Thus, they are suitable for many different approaches (Reid et al., 2011). The safety of live microorganisms persists as a debated issue, especially for susceptible individuals (Ravel et al., 2011; Borges et al., 2014). For this reason, different strategies have been developed to replace the use of live microorganisms, such as the use of killed probiotics called paraprobiotics (Trindade et al., 2021), microbial extracts, and cell-free supernatants (Piqué et al., 2019; Batista et al., 2022). Thus, antimicrobial elements secreted by probiotic microorganisms can be called “postbiotics,” which include non-viable cells that promote health benefits to the host when administered in adequate amounts (Collado et al., 2019); Salminen et al., 2021).

Postbiotics are compounds from several bioactive compounds that are secreted by the action of microorganisms either on food ingredients (e.g., bioactive peptides) or during growth and fermentation in complex microbiological cultures having bioactive soluble factors (Aguilar-Toalá et al., 2018; Collado et al., 2019; Barros et al., 2020; Cuevas-González et al., 2020). Organic acids, short-chain fatty acids, carbohydrates, antimicrobial peptides, enzymes, vitamins, cofactors, immune-signalling compounds, and complex agents have also been described as bioactive metabolites present in postbiotics prepared from lactic acid bacteria (LAB) (Moradi et al., 2019; Rajakovich and Balskus, 2019).

Exciting features of postbiotics, such as no biogenic amine production, a safe structure, being incapable of transferring antibiotic resistance, a defined chemical composition, stability in a broad range of temperatures and pH, and a broad spectrum of antimicrobial activity, make them valuable ingredients to be used in different approaches (Barros et al., 2020; Rad et al., 2021).

In this context, we aimed to develop an interesting and alternative use of the postbiotics of four LAB with potential probiotic characteristics (*Lactiplantibacillus plantarum*, *Lactobacillus acidophilus*, *Lactobacillus johnsonii*, and *Lactobacillus delbrueckii*) to combat *P. aeruginosa* and their ability to reduce the formation of biofilms by this pathogenic bacteria.

## 2 Materials and methods

### 2.1 Bacterial strains and growth conditions

The bacterial strains used in this study are summarised in Table 1. The four probiotic strains belonging to the Lactobacilli group (*L. acidophilus*, *L. delbrueckii*, *L. johnsonii*, and *L. plantarum*) were grown in De Man, Rogosa, and Sharpe agar (MRS, neogem®, Rüdersdorf, Germany) for 24 h at 37°C. *L. acidophilus* and *L. plantarum* were isolated from a typical fermented Mexican drink; *L. delbrueckii* was isolated from raw cow’s milk from the culture collection of the Centro de Investigación y Desarrollo en Criotecnología de Alimentos (CIDCA, Facultad de Ciencias Exactas, Universidad Nacional de La Plata, Argentina); and *L. johnsonii* was isolated from a human vagina and kindly provided by Dr. Regina Nardi Drummond from the Laboratório de Microbiologia Aplicada of the Departamento de Microbiologia at the Universidade Federal de Minas Gerais (UFMG).

**TABLE 1:** Bacteria strains and precedence.

Strains	Origin
*Pseudomonas aeruginosa* 27853™ (ATCC^®^)	American Type Culture Collection (ATCC^®^)
*Pseudomonas aeruginosa* 9027™ (ATCC^®^)	American Type Culture Collection (ATCC^®^)
*Lactobacillus delbrueckii* CIDCA 133	Centro de Investigación y Desarrollo en Criotecnología de Alimentos (CIDCA, Facultad de Ciencias Exactas, Universidad Nacional de La Plata, Argentina)
*Lactobacillus acidophilus*	Laboratório Nacional de Nutrigenómica y Microbiómica Digestiva Animal, Morelia, Mexico
*Lactobacillus plantarum*	Laboratório Nacional de Nutrigenómica y Microbiómica Digestiva Animal, Morelia, Mexico
*Lactobacillus johnsonii*	Laboratório de Microbiologia Aplicada of Universidade Federal of Minas Gerais (UFMG), Brazil

A single colony from each strain was transferred into the MRS broth under the same incubation conditions for 24 h to obtain the cell-free spent medium (CFSM). Pathogenic *P. aeruginosa* strains from the American Type Culture Collection (ATCC^®^) (PA 9027™ and PA 27853™) were cultured in the brain heart infusion (BHI) medium (HiMedia, India) for 16 h at 37°C.

### 2.2 Preparations of cell-free spent medium of probiotic strains—postbiotic preparation

#### 2.2.1 Growing medium

The active overnight culture was diluted in fresh medium, starting from an initial optical density (OD_600nm_) of 0.04, and incubated at 37°C in microaerobiosis for 24 h. To create this condition, the falcon tubes were filled to the top with the medium without shaking for 24 h at 37°C. The CFSM of each probiotic strain was obtained as previously described by Bayoumi and Griffiths (2012). Briefly, the overnight culture of the four probiotic strains was prepared by centrifugation at 6,000*g*/4°C for 10 min, and the cells were then removed. The supernatant was filter sterilised with a 0.22 μm pore filter (Kasvi, São José dos Pinhais, Paraná) to remove the residue of viable cells and referred to as CFSM. An aliquot of the CFSM of each probiotic strain was plated on solid MRS medium and incubated at 37°C for 48 h to ensure the absence of viable cells. The CFSM of all probiotic strains was stored at −20°C until use in further assays.

### 2.3 Antibacterial activity of CFSM in planktonic condition

#### 2.3.1 Determination of minimum inhibitory concentration and minimum bactericidal concentration

To determine the minimum inhibitory concentration (MIC) of each CFSM, the broth microdilution method was used in 96-well round-bottomed polystyrene microtiter plates (SPL Life Sciences, Pocheon-si, Korea) conducted according to the methodology described by Ruíz et al. (2012), with some modifications. The CFSM was serially diluted in the BHI broth (HiMedia, India) using a two-fold dilution; 100 μL of diluted CFSM was placed in each well and 25 μL of an overnight-grown culture of the indicator strain, *P. aeruginosa*, was added after adjusting its absorbance to 0.5 at 600 nm optical density equivalent to 3.0 × 10^14^ colony-forming units (CFU)/mL for PA 27853™ and 14.8 × 10^14^ CFU/mL for PA 9027™. The plate was incubated at 37°C for 24 h under aerobic conditions. Positive (*P. aeruginosa* + BHI) and negative (only BHI medium) controls were also included on the plate. The MIC values were defined as the lowest concentration of CFSM that could inhibit the visible growth of the pathogenic bacteria.

To assess the minimum bactericidal concentrations (MBCs), 50 μL from the wells with no apparent growth, and without turbidity, were spread onto the surface of solid BHI agar (1.5%) (BD Difco™ Bacto agar, United States) on Petri dishes and incubated at 37°C for 24 h. These assays were performed as three independent experiments with three replicates per CFSM analysed, and the experiment was conducted at three different times. The sample with the lowest concentration of CFSM that showed no growth on BHI agar was recorded as the MBC. The MIC levels were further used in the following assessment.

#### 2.3.2 Antibacterial activity

##### 2.3.2.1 Inhibition zone assay

The overnight cultures of *P. aeruginosa* (ATCC^®^ 9027™ and 27853™) were cultured in BHI medium for 16 h at 37°C. A suspension of each bacterium of 0.5 absorbance (OD_600nm_) was adjusted for use in the agar diffusion method to determine the growth inhibition zone of the tested bacteria, following the methodology as described by Cruz et al. (2001), with some modifications. First, a bottom layer (9 mL) of MRS was prepared in the Petri dish (90 × 60 mm). Second, a top layer of molten and cooled BHI medium (5 mL) was mixed with each tested bacterial suspension, containing 3.0 × 10^14^ and 14.8 × 10^14^ CFU/mL for PA 27853™ and 9027™, respectively, and poured onto the bottom layer. One well of 9 mm diameter was prepared in each plate, and 100 μL (1×), 200 μL (2×), and 300 μL (3×) of each pure postbiotic were introduced into each well. The plates were incubated for 24 h at 37°C. A clear inhibition zone of more than 1 mm was formed around the well and measured and recorded as a positive inhibitory result. The measurement was performed from the edge of the formed halo. The experiment was conducted at three different times. To evaluate whether the potential inhibitory effect of the CFSM could be due to its acidic pH, it was neutralised (pH = 7) with NaOH (5 M) (Neon, São Paulo, Brazil), and the assay was repeated twice.

##### 2.3.2.2 Antibacterial activity of CFSM *Lactobacillus* supernatants against *Pseudomonas aeruginosa* in planktonic cultures

The antibacterial activity of the CFSM for four Lactobacillaceae strains against planktonic cultures of *P. aeruginosa* (PA 9027™ and PA 27853™) was assessed according to the methodology described by Lin et al. (2015), with some modifications. Briefly, the CFSM was prepared as described previously; the overnight culture of the microbial cells was centrifuged (5,000 rpm/5 min), and the pellets were washed twice with 0.90% NaCl solution (Synthia, Sao Paulo, Brazil). As mentioned above, the cell suspensions were adjusted to 0.5 (OD_600nm_). Then, 250 μL of each *P. aeruginosa* suspension was faced with 250 μL each of the CFSM and mixed with 1.5 mL of sterile physiological solution. The control group was formed as follows: 250 μL of microbial *P. aeruginosa* suspension was faced with 250 μL of MRS medium (the medium where the probiotic bacteria were grown) plus 1.5 mL of sterile physiological solution. The cultures were incubated at 37°C for 24 h and then diluted and plated on a solid BHI medium (HiMedia, India). The plates were incubated (37°C/24 h), and the CFU/mL was determined. This assay was performed as two independent experiments with two replicates per group.

### 2.4 *Pseudomonas aeruginosa* growing at increased CFSM concentrations

To establish the percentage of CFSM able to inhibit the growth of *P. aeruginosa*, the growth curve assay was conducted with increased CFSM concentrations. This assay was performed as two independent experiments. Briefly, 200 μL of overnight culture at 0.04 (OD_600nm_) (8.3 × 10^9^ CFU/mL for PA 9027™ and 7.43 × 10^9^ CFU/mL for PA 27853™) was placed in 96-well polystyrene plates (SPL Life Sciences, Pocheon-si, Korea) as the initial point of the curve, this being the point with 100% of pathogen cells. Then, 98% of the pathogen culture (196 μL) was placed in the next well with 2% (4 μL) of each CFSM; 96% of the pathogen culture and 4% of each CFSM were placed in the next well; and so on, with the final well containing 40% of the pathogen cells and 60% of each CFSM. The microtiter plates were incubated at 37°C for 16 h when the pathogen strains had reached the stationary phase. The optical density was then measured. This assay was performed in triplicate. It is important to highlight that the same experiment was previously carried out with pathogens and increasing MRS broth volumes to evaluate if the MRS had any influence on the growing pathogens.

### 2.5 Determination of the anti-adhesion activity of CFSM from *Lactobacillus* strains against *Pseudomonas aeruginosa* spp. biofilm

The anti-adhesion activity of the CFSM for the Lactobacillaceae against *P. aeruginosa* spp. was performed in pre-coated, co-incubated, and preformed biofilm experiments.

#### 2.5.1 Pre-coated experiments

The pre-coated experiments were carried out according to the methodology described by Gudiña et al. (2010). Briefly, 96-well microtiter plates (SPL Life Sciences, Pocheon-si, Korea) were coated with 200 μL of different CFSMs in triplicate, and the microtiter plates were incubated at 37°C for 24 h. This volume was chosen to cover both the bottom and sides of the well. After this time, the CFSM was removed, and plates were washed twice with 100 μL of phosphate buffer saline (PBS, 0.1 M), pH 7.2, to remove the non-adhering supernatant. Then, 200 μL of each 16-h growth culture of *P. aeruginosa* suspension (1 × 10^9^ CFU/mL) cultured in BHI broth (HiMedia, India) with sucrose (1%) to enhance the formation of densely clustered microcolonies in polystyrene material (LabSynth, São Paulo, Brazil) was added to each well. The microtiter plate was incubated at 37°C for 24 h under sterile conditions to allow cell attachment. To remove the non-adhering cells, the plate was gently washed twice with 200 μL PBS (0.1 M), pH 7.2. Biofilm formation was assessed using the Crystal Violet assay (Newprov, Pinhais, Paraná, Brasil). This methodology was chosen as the reference protocol for detecting biofilm formation by isolated food pathogens (Rodrigues et al., 2010; Dias-Souza, 2013; Marinho et al., 2013). The formed biofilm was fixed for 15 min by adding 300 μL of methanol (99% v/v) (Dinâmica, São Paulo, Brasil) to each well, and the plate was air dried. After that, 300 μL of crystal violet (0.5%) was added and incubated at room temperature for 15 min. Crystal violet was then removed, the wells were washed with 200 μL of distilled water to remove the excess stain, and the plate was air dried. Then, 200 μL of 95% ethanol (Dinâmica, São Paulo, Brasil) was added to the wells, and 100 μL of this distaining solution from each well was transferred to a new plate. The absorbance was then measured at 595 nm using a microplate reader (Multiskan Spectrum, Thermo Scientific). Pathogenic bacterial suspension without CFSM was prepared as the control. The percentage reduction in adherence was calculated using the following equation:

% microbial adhesion = 1 − (ODc/ODo) × 100

where ODc represents the optical density of the well with CFSM and pathogenic suspension, while ODo represents the optical density of the pathogenic suspension without CFSM (control).

The microtiter plate anti-adhesion assay estimates the percentage reduction of pathogenic bacteria adhesion to the control wells, which was 0% in the absence of Lactobacillaceae CFSM. This analysis was carried out in triplicate, and the mean optical density was taken.

#### 2.5.2 Co-incubated/inhibition of initial cell attachment

In the co-incubated experiment, the capacity of the CFSMs to inhibit biofilm formation was evaluated. The suspension of the previously activated pathogenic bacteria (*P. aeruginosa*) was centrifugated at 10,000*g* for 5 min. The supernatant was discarded, and the pellet was washed thrice with PBS (0.1 M), pH 7.2. The cells were resuspended in BHI broth (HiMedia, India) with sucrose 1% (LabSynth, São Paulo, Brazil) and adjusted to absorbance 0.5 (OD_600nm_) equivalent to 3.0 × 10^14^ CFU/mL for PA 27853™ and 14.8 × 10^14^ CFU/mL for PA 9027™. Then, 100 µL of each bacterial solution was added to the individual well of a sterile flat-bottomed 96-well polystyrene microtiter plate (SPL Life Sciences, Pocheon-si, Korea) with 100 μL of different Lactobacillaceae CFSMs, and the plate was incubated at 37°C for 24 h. For the negative control, only BHI + sucrose 1% medium was added without the bacterial suspension, and the positive control was the bacterial suspension without CFSM. Biofilm formation was assessed using the Crystal Violet assay as described above. The mean absorbance (OD_595nm_) was used to determine the percentage inhibition of biomass formation according to the following equation:

Percentage inhibition = 100 − [(OD_595nm_ experimental well with CFSM/OD_595nm_ control well without CFSM) × 100].

#### 2.5.3 Inhibition of preformed biofilm and evaluation of biomass attachment

The effect of the CFSM on biofilm growth and development was evaluated as described by Jadhav et al. (2013). Biofilms were allowed to form for 24 h before adding the CFSM. Biofilm formation was achieved by transferring 100 μL of bacterial culture that was prepared as described above (OD_600nm_ = 0.5) into the wells of a sterile flat-bottomed 96-well polystyrene microtiter plate in triplicates. The microtiter plates were covered and incubated for 24 h at 37°C to allow cell attachment and biofilm formation. Following incubation, 100 μL of each CFSM was added to each well to reach a final volume of 200 µL. The plates were then incubated for 24 h. As the negative control, BHI + sucrose 1% medium was added without bacterial suspension, and the positive control was the bacterial suspension without CFSM. Finally, the biofilms were assessed for biomass attachment using the Crystal Violet assay, as previously mentioned. The Crystal Violet assay was performed as three independent experiments with n = 6 biofilms per CFSM.

### 2.6 Biofilm metabolic activity assay

To assess the metabolic activity of the biofilm formed, the modified 3-[4,5-dimethylthiazol-2-yl]-2,5-diphenyltetrazolium bromide (MTT) reduction assay was used according to the study by Schillaci et al. (2008). MTT salt (Sigma Aldrich, São Paulo, Brazil) was dissolved in 0.1 M PBS to give a final concentration of 5 mg/mL. Plates containing preformed biofilm were incubated with 100 μL of each CFSM for 24 h. Then, the medium was gently removed, the plates were air dried, and 100 μL of MTT solution was added to each well and incubated for 3 h at 37°C under sterile conditions. The insoluble purple formazan (obtained by enzymatic hydrolysis of MTT by the dehydrogenase enzyme found in living cells) was further dissolved in 100 μL of dimethyl sulphoxide (DMSO, Sigma Aldrich, Darmstadt, Germany). The absorbance was then measured at an optical density of 570 nm using a microplate reader (Multiskan Spectrum, Thermo Scientific). The MTT assay was performed as three independent experiments with n = 6 biofilms per CFSM.

### 2.7 Analysis of biofilms by scanning electron microscopy

SEM analysed the effect of each postbiotic on biofilm inhibition. The goal was to use this technique to show biofilm inhibition by the CFSM. We chose only the co-incubated conditions of two *P. aeruginosa* studies since this is a qualitative experiment. To develop this assay, we used glass slides (0.13–0.17 mm thick, Global Glass, China) following the methodology as described by Marttinen et al. (2013) and Rossoni et al. (2018), with some modifications. The sterilised slides were placed in a 24-well polystyrene plate (SPL Life Sciences, Pocheon-si, Korea), and 400 μL of the overnight bacterial culture that was prepared as described above (OD_600nm_ = 0.5) was added. The plates were incubated in a bacteriological incubator at 37°C for 1 h to promote the initial adhesion of *P. aeruginosa* onto the discs. Afterwards, 400 μL of each CFSM was added to each well. For the positive control, 400 μL of the MRS medium was used instead of the CFSM; the negative control was BHI + 1% sucrose medium, without slides. The plates were incubated at 37°C for 16 h. After biofilm formation, the wells were washed twice with 0.1 M PBS, and the specimens were fixed with methanol (99%) (Dinâmica, São Paulo, Brasil) for 1 h. The samples were then dehydrated in increasing ethanol (Dinâmica, São Paulo, Brasil) concentration series (10%, 25%, 50%, 75%, and 90%) for 20 min each, followed by immersion in 100% alcohol for 1 h. The plates were kept in a bacteriological incubator at 37°C for 24 h to permit total drying of the specimens. The dried slides were transferred to aluminium stubs and sputter coated with gold for 160 s at 40 mA (Denton Vacuum Desk II, Denton Vacuum LLC, Moorestown, NJ, United States). The samples were examined and imaged using a JEOL JSM-5600 scanning electron microscope (JEOL United States, Inc., Peabody, MA, United States) at the Characterisation Laboratory, Materials Engineering Department, Centro Federal de Educação Tecnológica de Minas Gerais (CEFET-MG). These experiments were performed twice with n = 2 biofilms per group.

### 2.8 Statistical analysis

The effect of each CFSM on biofilm formation was analysed using the GraphPad Prism 9.1 software. The one-way analysis of variance (ANOVA) method was used for multiple comparisons, followed by Tukey’s post hoc test (parametric data) or by the Kruskal–Wallis test and post-tested by using the Dunn’s test (non-parametric data). All experiments were performed at least in duplicates, and the standard deviation from the mean was calculated. Values were considered significantly different from each other at *p* < 0.05.

## 3 Results

### 3.1 CFSM presented different MICs and MBCs for *Pseudomonas aeruginosa* (27853™ and 9027™)

The MIC values of different CFSMs were determined by the microdilution method, and the results varied depending on the pathogenic bacteria and CFSM tested. The MIC and MBC values are presented in Table 2 *.* The MIC values of each CFSM are important to investigate the mechanism of action of these products for their ability to inhibit biofilms. Pure CFSM and its concentrated forms (1×, 2×, and 3×) were used to analyse the planktonic growth and inhibition zones.

**TABLE 2:** MIC and MBC values.

LAB strain	PA 9027™		PA 27853™	
	MIC	MBC	MIC	MBC
*L. acidophilus*	½	1	¼	½
*L. delbrueckii*	¼	1	¼	1
*L. johnsonii*	¼	1	1/8	¼
*L. plantarum*	¼	-	¼	-

1 indicates pure CFSM, without any dilution; — indicates not found in the tested concentration.

### 3.2 Inhibition of planktonic *Pseudomonas aeruginosa* spp. by CFSM and growth analysis

**FIGURE 1:**
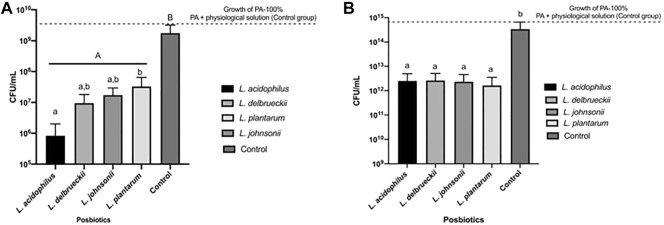
Growth expressed by *P. aeruginosa* obtained by counting the CFU/mL in the antibacterial activity *in vitro* test for 24 h in the presence of *Lactobacilli* supernatants. The supernatants of four *Lactobacilli* strains were tested. **(A)** Graph of 100% growth of PA 2853™ (1.71 ± 1.44 × 10^9^ CFU/mL) and the CFU/mL of PA in the presence of different *Lactobacillus* supernatant. **(B)** Graph of 100% growth of PA 9027™ (3.05 ± 3.18 × 10^14^ CFU/mL) and the CFU/mL of PA in the presence of different CFSMs. Different capital letters refer to significant differences (*p* < 0.05) between treatments and the control group by ANOVA followed by Tukey’s *post hoc* test. Different lowercase letters indicate statistically significant differences (*p* < 0.05) between the tested treatments by ANOVA followed by Tukey’s *post hoc* test.

To evaluate whether the MRS broth of Lactobacillaceae culture interferes with the results, we included a control group with only *P. aeruginosa* and the MRS medium. The same experiment was conducted using a physiological solution instead of the BHI medium and using the MRS as control. The obtained results showed no interference in *P. aeruginosa* growth with the medium used to prepare the CFSM, as shown in Figures 1A, B. The four CFSMs analysed presented antibacterial activity against both tested strains of *P. aeruginosa*. A reduction in the number of CFU/mL of pathogenic cells was observed when they were faced with the CFSMs when compared to the pathogens that faced physiological solution, the control group (100% *P. aeruginosa* growth means 1.71 × 10^9^ ± 1.44 × 10^9^ CFU/mL for PA 27853™ and 3.05 × 10^14^ ± 3.18 × 10^14^ for PA 9027™). The observed reduction, expressed in percentage, to PA 27853™ growth was 99.98% with CFSM from *L. acidophilus*, 99.47% from *L. delbrueckii*, 98.19% from *L. plantarum*, and 98.83% from *L. johnsonii*; and the observed reduction to PA 9027™ was 99.28% for CFSM from *L. acidophilus*, 99.14% from *L. delbrueckii*, 99.66% from *L. plantarum*, and 99.29% from *L. johnsonii*. All the samples were statistically different from the control (*p* < 0.001), yet similar.

### 3.3 CFSM can stop growth of pathogen PA successfully

**FIGURE 2:**
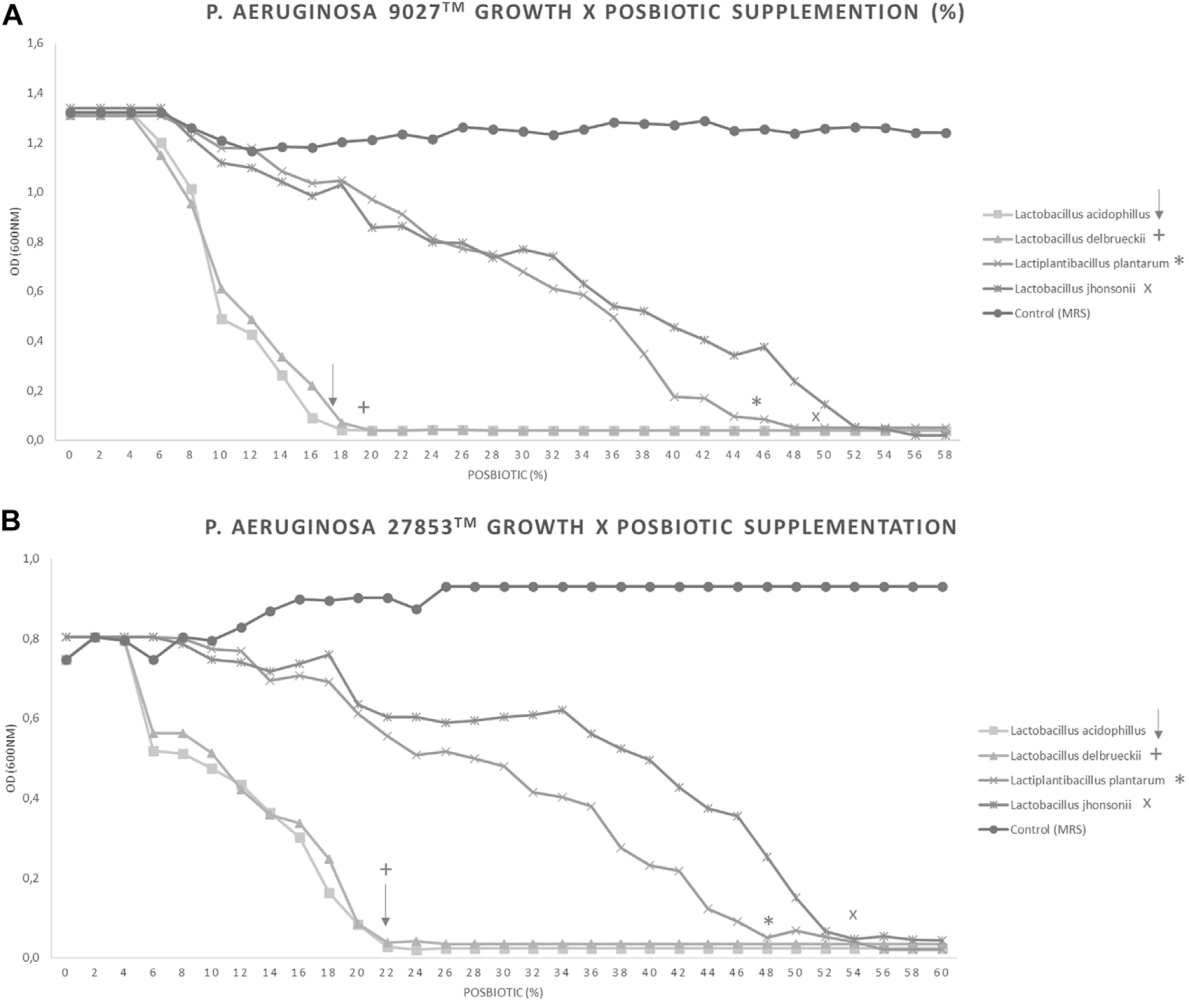
. Different percentages of CFSM supplementation can inhibit the growth of two *Pseudomonas aeruginosa* strains. Increasing amounts of each CFSM were added to the medium containing *P. aeruginosa*. CFSMs tested against PA 9027™ **(A)** and PA 27853™ **(B)**. Symbols show the percentage of total inhibition of growth achieved: ↓ CFSM from *L. acidophilus*, + CFSM from *L. delbrueckii*, * CFSM from *L. plantarum*, and × CFSM from *L. johnsonii*.

The inhibition of *P. aeruginosa* growth was evaluated in the presence of different percentages of CFSM in the four studied strains at 16 h. An absorbance (OD_600nm_) of 0.04 or less means that there was no growth or that it was very low. This result obtained for PA 9027™ with 18%, 20%, 46%, and 50% of *L. acidophilus*, *L. delbrueckii* CIDCA 133, *L. plantarum*, and *L. johnsonii* CFSM, respectively, is shown in Figure 2A. For PA 27853™, 22%, 22%, 48%, and 54% of *L. acidophilus*, *L. delbrueckii* CIDCA 133, *L. plantarum*, and *L. johnsonii* CFSM, respectively, showed an absorbance (OD_600nm_) of 0.04 (Figure 2B). These results demonstrate the inhibitory power of different CFSMs with respect to *P. aeruginosa* growth.

### 3.4 CFSM can produce inhibition zone in *Pseudomonas aeruginosa* growth

The tested CFSMs could inhibit both strains of PA in an inhibition zone ranging between 3.00 and 7.00 mm, as shown in Table 3. Considering the maximum concentration tested (300 μL = 3×), our results demonstrated that the highest antibacterial activity could be attributed to *L. acidophilus* CFSM, which showed inhibition zones of 5.67 ± 1.15 mm and 7.00 ± 2.64 mm for PA 9027™ and PA 27853™, respectively. Although *L. johnsonii* CFSM was the postbiotic with a lower inhibition zone, 3.00 ± 1.00 mm and 3.33 ± 1.53 mm for PA 9027™ and 27853™, respectively (Table 3), the inhibition zone was significant.

**TABLE 3:** Antibacterial activity at different concentrations of CFSM against two *Pseudomonas aeruginosa* strains

	Strains (CFSM)	PA 9027™			PA 27853™		
		Inhibition zone (mm)			Inhibition zone (mm)		
		100 μL (1×)	200 μL (2×)	300 μL (3×)	100 μL (1×)	200 μL (2×)	300 μL (3×)
	*L. acidophilus*	0	3.5 ± 0.71	5.67 ± 1,15	0	6.00 ± 1.31	7.00 ± 2.65
	*L. delbrueckii*	0	3.5 ± 0.71	5.33 ± 1.15	0	5.00 ± 0.89	5.67 ± 1.15
	*L. plantarum*	0	2.5 ± 0.71	4.67 ± 0.58	0	0.5 ± 0.93	3.09 ± 1
	*L. johnsonii*	0	0	3.00 ± 1.00	0	0	2.00 ± 2.12

### 3.5 Reduction of biofilm formation in pathogenic *Pseudomonas aeruginosa* by CFSMs

**FIGURE 3:**
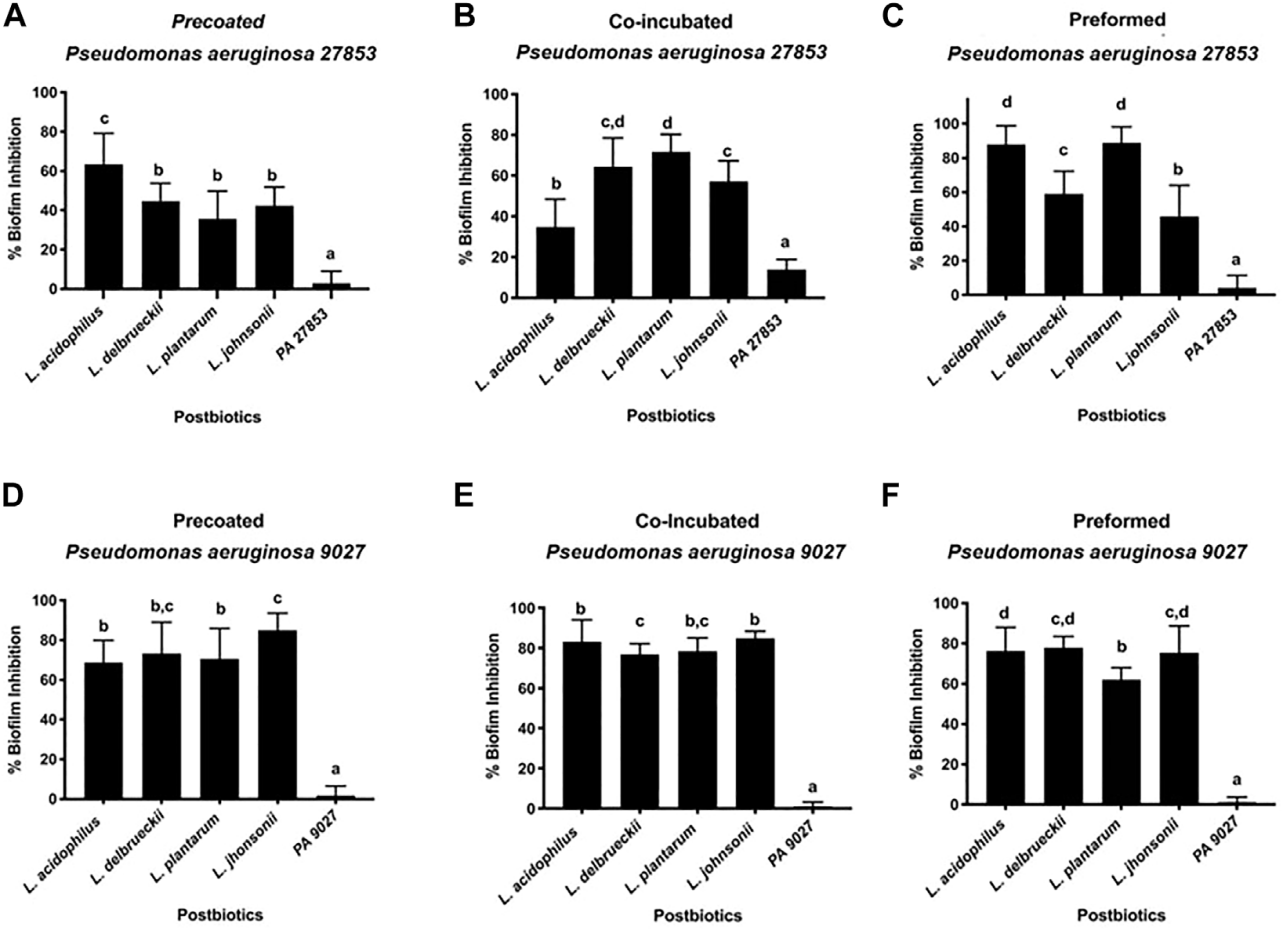
Effect of different CFSMs expressed as a percentage of inhibition of biofilm formation, on pre-coated **(A, D)**, co-incubated **(B, E)**, and preformed **(C, F)** biofilms on two Pseudomonas aeruginosa strains (27853™ - **A–C** and 9027™ **D–F**).

To confirm how the CFSM of different Lactobacillaceae could influence the extracellular matrix of *P. aeruginosa* biofilms, the total biomass was quantified by colourimetric assay using crystal violet. This assay is used as an indicator of cellular adhesion, either for viable or non-viable attached cells (Kouidhi et al., 2010). As PA 27853™ and PA 9027™ have a high ability to produce biofilms, their ability to decrease biofilm formation was tested in three different conditions when assessed at the MIC concentration of CFSM: 1) the initial cell attachment by planktonic cells (pre-coated), 2) co-incubation (planktonic cells associated with CFSM), and 3) on preformed (24 h) biofilms. These assays indicate a positive effect on biofilm inhibition for all CFSMs that were tested for PA 27853™ or PA 9027™. For PA 27853™, the high percentage of inhibition in a pre-coated approach was represented by the CFSM from *L. acidophilus* (63.457% ± 15.746%), which showed statistical differences (*p* < 0.05) from the other CFSM (44.742% ± 8.991% for *L. delbrueckii*, 35.750% ± 13.920% for *L. plantarum*, and 42.370% ± 9.439% for *L. johnsonii*) and the control (Figure 3A). The co-incubation experiment exposed over 50% biofilm inhibition by the CFSM of *L. delbrueckii* (64.352% ± 3.116%), *L. plantarum* (71.705% ± 2.074%), and *L. johnsonii* (57.154% ± 2.821%); nevertheless, *L. acidophilus* showed 34.768% ± 3.537% of biofilm inhibition (Figure 3B). Preformed biofilms were significantly reduced in the presence of CFSM from *L. acidophilus* (88.070% ± 10.652%) and *L. plantarum* (88.841% ± 9.304%). Although CFSM from *L. delbrueckii* (59.140% ± 13.096%) and *L. johnsonii* (45.822% ± 18.181%) had a lower inhibition of preformed biofilm, it was still significant (Figure 3C). Regarding the crystal violet results of PA 9027™, in the three studied approaches, it was possible to observe that the *L. johnsonii* CFSM had a high percentage of biofilm inhibition, that is, 84.925% ± 8.701%, 84.872% ± 3.510%, and 75.509% ± 13.227% for pre-coated, co-incubated, and preformed approaches, respectively. Although the other CFSM studies presented a lower percentage of inhibition, they still had a high and significant inhibition rate of up to 62% in the three methodologies used (Figures 3D–F).

**FIGURE 4:**
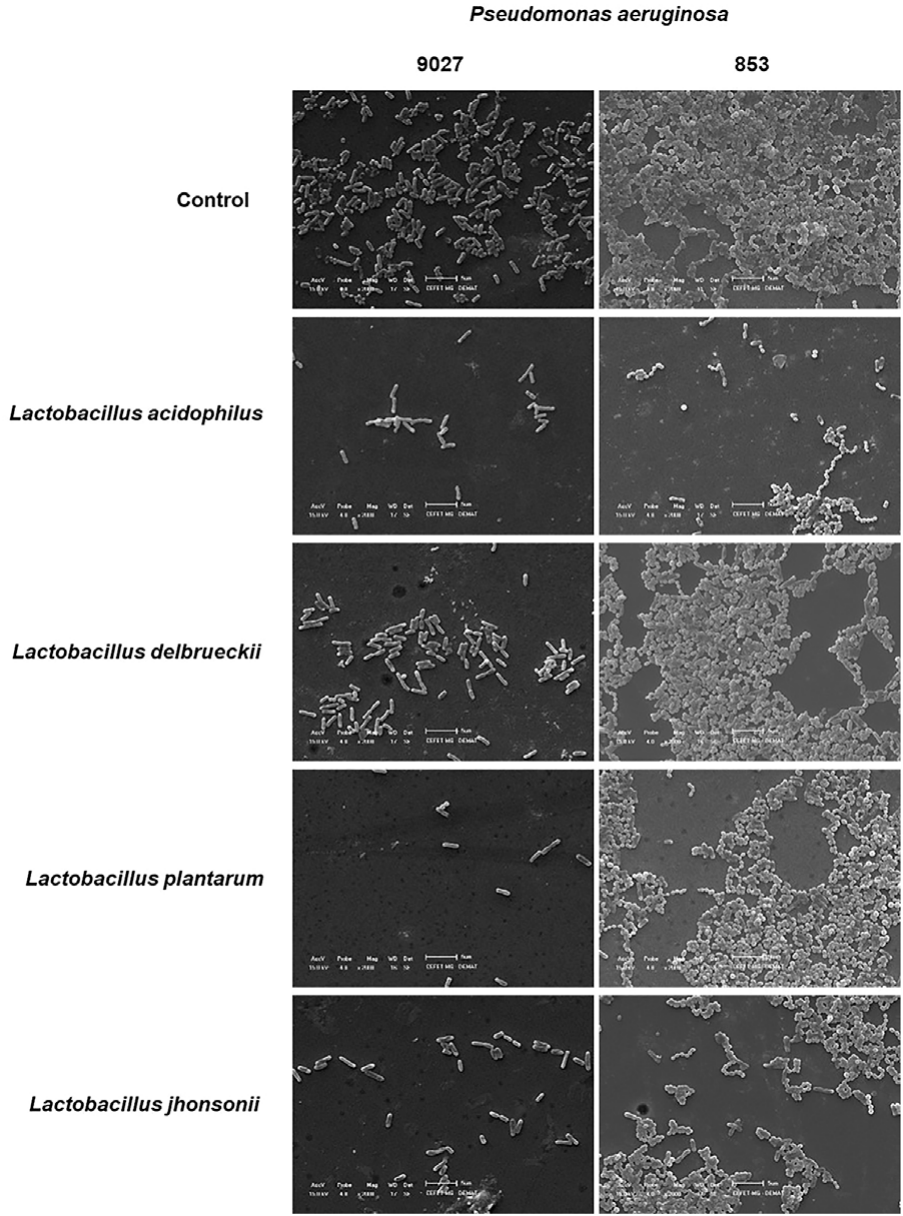
SEM of biofilm formation and inhibition *in vitro* assay. The images of the control groups of PA 27853™ and 9027™ in the BHI medium (sucrose, 10%) present numerous bacterial cells and biofilm formation. The magnification used for the image was ×2,000 using a JEOL JS-5600 scanning electron microscope (JEOL United States, Inc., Peabody, MA, United States) at the Characterisation Laboratory, Materials Engineering Department, Centro Federal de Educação Tecnológica de Minas Gerais (CEFET-MG). It was possible to observe a reduction in the number of PA cells and their aggregation in the CFSM treatments when compared to the respective controls.

The biofilm was also evaluated by SEM analysis, in which either PA 27853™ or PA 9027™ showed strong adherence on the glass slides and a compact island-like biofilm covered by a large amount of dense white network-like structure. By this technique, it is possible to qualitatively verify the reduction in the number of PA cells and also a reduction in the cell’s compaction and a drop in or the absence of the dense white network-like structure, which represents the inhibitory effect of the CFSM in PA biofilm formation (Figure 4) when the control biofilm is compared with the biofilm that is exposed to the CFSM.

### 3.6 CFSM presented reduced metabolic activity in remnant biofilm

**FIGURE 5:**
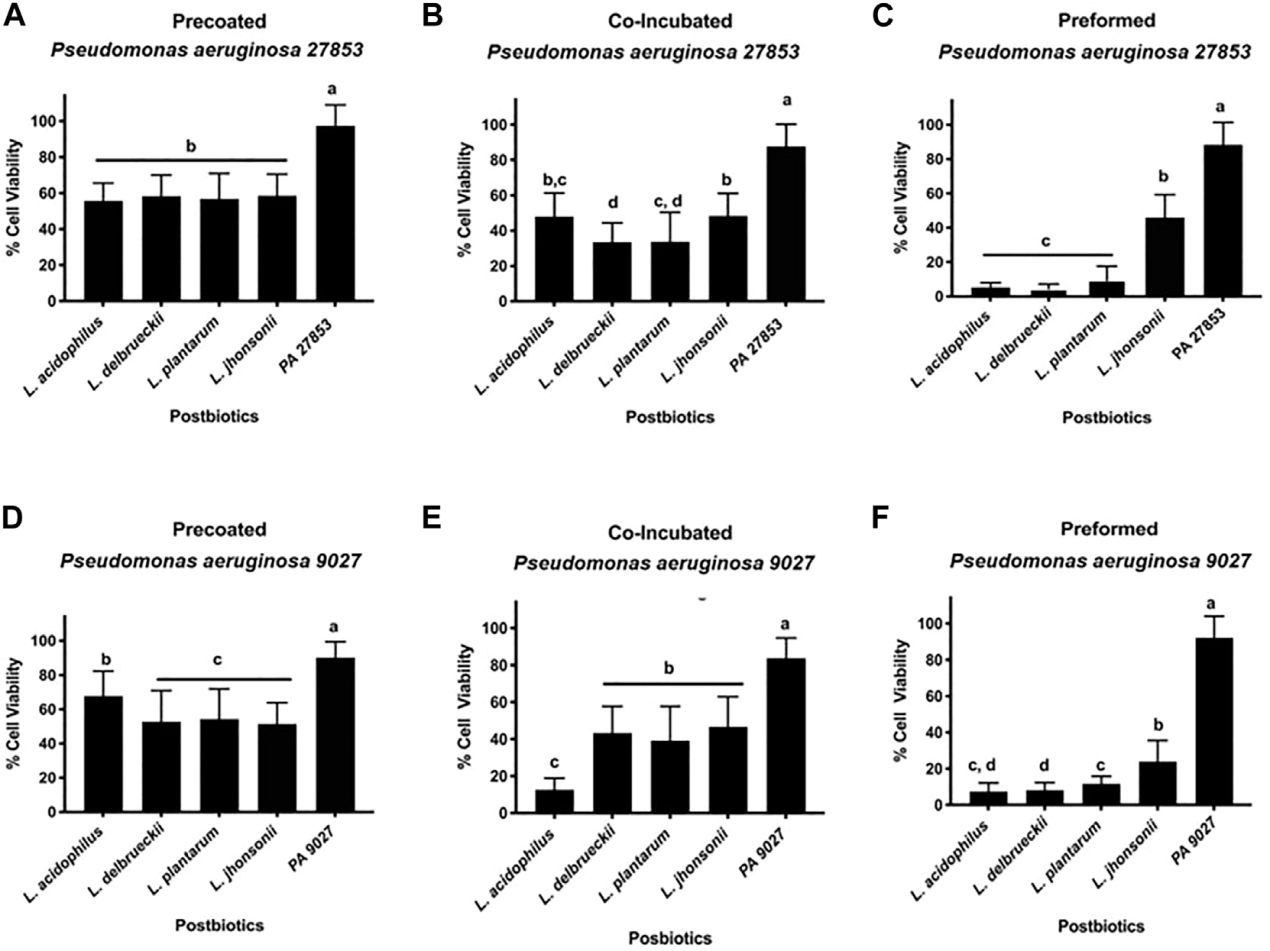
**(A–F)** Effect of CFSMs on the metabolic activity of pre-coated **(A, D)**, co-incubated **(B, E)** and preformed **(C, F)** biofilm cells of Pseudomonas aeruginosa 27853™ **(A–C)** and 9027™ **(D–F)** incubated for 24 h was determined by MTT performance. Results show that the metabolic activity of CFSM in treated cells was reduced when compared to the untreated control. Different letters (a, b, c, and d) indicate statistically significant differences (*p* < 0.05) by ANOVA followed by Tukey’s *post hoc* test.

The correlation between the Crystal Violet and MTT assay results provides a better understanding of the influence of the different CFSMs tested on biofilm inhibition. The MTT assay is an indicator of cell viability (Krom et al., 2007) because it describes the attached cells' metabolic status. Thus, the MTT assay is performed to confirm the viability of cells present in the different approaches in biofilm formation. The experimental controls, PA 27853™ and PA 9027™, showed increased metabolic activity, as expected, with percentages of up to 80% for both pathogenic strains. The MTT results for PA 27853™ showed cell viability of approximately 55% for all CFSMs tested in the pre-coated biofilm, and there were no significant differences between the different CFSMs; nevertheless, there were substantial differences with the control (Figure 5A). In the co-incubated condition, 33.490% ± 10.867% cell viability was observed in the formed biofilm in the presence of the CFSM for *L. delbrueckii*, 33.663% ± 10.867% for *L. plantarum*, 47.836% ± 13.470% for *L. acidophilus*, and 48.320% ± 12.820% for *L. johnsonii*, statistically differentiating these from the positive control, which showed a cell viability of approximately 90% (Figure 5B). In the preformed biofilm treated with postbiotics from *L. acidophilus*, *L. delbrueckii*, and *L. johnsonii*, this pathogenic strain presented the lowest cell viability of 5.252% ± 2.735%, 4.399% ± 2.879%, and 9.420% ± 8.145%, respectively. Even though the CFSM from *L. johnsonii* manifested the highest cell viability (45.904% ± 13.454%), it was still statistically different from the control (Figure 5C).

The cell viability presented with PA 9027™ in the pre-coated biofilm was approximately 50% for the CFSM of *L. delbrueckii*, *L. plantarum*, and *L. johnsonii* without statistical differences between them (*p* > 0.05). In this approach, *L. acidophilus* was the postbiotic that showed the highest cell viability (67.715% ± 14.487%); however, it was lower than that of the control, which presented a cell viability of 88.307% ± 12.906%, with a statistical difference of *p* < 0.05 (Figure 5D). Contrary to this result, for the co-incubation conditions, the CFSM from *L. acidophilus* presented the lowest cell viability (12.622% ± 6.225%) being the only treatment that was statistically different from the other treatments that showed a cell viability between 39% and 46%; however, these treatments showed statistical differences when compared with the control group (83.771% ± 10.825%) (Figure 5E). Finally, as shown with PA 27853™ for the preformed biofilm, *L. johnsonii* was the CFSM that presented a high cell viability (23.871% ± 11.768%) when compared with the other CFSMs, which showed a cell viability of approximately 10% (Figure 5F).

## 4 Discussion

Postbiotics have gained more attention in the last decade due to their beneficial actions on the host without the adverse risk of inducing bacteraemia in immunocompromised patients from the delivery of live cells (Gao et al., 2019). Consequently, the works of several research groups have produced satisfactory results for this biotic fraction.

*Pseudomonas aeruginosa* is one of the most significant bacteria that can cause enormous burden to public health, having the capacity to adapt its genome and physiology throughout chronic infections and develop rapid antibiotic resistance; this pathogen has been listed in the global priority pathogens list by the World Health Organization (2017) and is considered an opportunistic pathogen (Cai et al., 2015). Controlling the growth of pathogens is as crucial as their elimination. Interference in their pathogenic mechanisms to regulate the production of bacterial virulence, such as in biofilm formation through the attenuation of the bacterial QS signalling system, by QS target agents (Jakobsen et al., 2012; Chen et al., 2019) is a necessary approach to delay the development of their resistance (LaSarre and Federle, 2013). For this reason, the present study was conducted to elucidate the antibacterial and antibiofilm potential of four supernatants, free of live cells, produced by four LAB against *P. aeruginosa*, one of the primary pathogens that effect infection of skin burns, delaying the healing process. Probiotics isolated from different niches display antibacterial effects against gram-negative and gram-positive microorganisms. The interest of researchers in studying the action of various cell-free supernatant probiotics is increasing due to the antibacterial properties assigned to the organic acids produced by these, which reduce the pH of the medium where these grow. In addition, it has been reported that the bioactive compounds released by probiotics, such as bacteriocins and hydrogen peroxide, are responsible for their antimicrobial properties (Noordiana, 2013; Yang et al., 2014; Drider et al., 2016). Thus, several research studies support the potential use of CFSMs and their bioactive compounds as antimicrobials against different pathogens.

Currently, “postbiotics” refer to soluble components with biological activity that could be a safer alternative to whole bacteria (Tsilingiri et al., 2012). The interest in postbiotics has increased in the last 5 years due to their beneficial actions on the host without the adverse risk of inducing bacteraemia, especially in immunocompromised patients and children, due to their capacity to deliver metabolites deprived of live cells (Gao et al., 2019).

The antimicrobial activity of drugs is usually assessed by the determination of the MIC and MBC of the drug *in vitro* after overnight aerobic incubation. Levison (2004) reported that the MBC is usually the same as the MIC for bactericidal drugs and generally not more than four-fold higher. On the contrary, the MBC of bacteriostatic medicines is multifold times more elevated than their MICs.

Considering the MIC and MBC results obtained for *P. aeruginosa*, it has been possible to show the bactericidal effect of CFSM from *L. acidophilus* and *L. johnsonii*. The other two CFSMs showed a bacteriostatic impact on PA 27853™. When these analyses were performed on PA 9027™, it was possible to attribute the bactericidal effect to the CFSM for *L. acidophilus* and the bacteriostatic effect to the rest of the CFSMs studied. Thus, all of the CFSMs of the tested Lactobacillaceae strains are potential agents against both *P. aeruginosa* strains. Our results are in accordance with the study by Saadatzadeh et al. (2013), who reported the powerful inhibitory effect of a cell-free extract of *L. casei* on *P. aeruginosa* and other pathogens that they tested, evidencing the anti-pathogenic impact of this biotic.

The antimicrobial susceptibility of PA was evaluated using agar overlay interference tests. It was shown that all Lactobacillaceae grown for 24 h on MRS medium formed a supernatant that exhibited inhibitory activity in the highest concentration tested (3×) for *P. aeruginosa*. It is essential to highlight that the CFSM of *L. acidophilus* showed an inhibition zone at a lower concentration for both *P. aeruginosa* 9027™ and 25853™, denoting the bactericidal effect of this CFSM. Meanwhile, *L. delbrueckii* presented bacteriostatic behaviour against both the tested strains of PA, showing an inhibition zone at the two highest concentrations tested. The CFSM of *L. plantarum* and *L. johnsonii* also showed an inhibition zone at the highest concentration tested. This antibacterial property of the CFSMs might be due to the presence of different organic acids in their composition since they have an acidic pH (of 3.70 for *L. acidophilus*, 4.2 for *L. delbrueckii*, 4.7 for *L. johnsonii*, and 4.7 for *L. plantarum*), and this antibacterial evidence can also be attributed to ethyl alcohol, short-chain fatty acids such as acetic acid (Bilkova et al., 2011), lactic acid, bacteriocins, hydrogen peroxide, and surfactants, which are some of the secondary metabolites produced by Lactobacilli that have antimicrobial activity (Noordiana, 2013; Yang et al., 2014; Drider et al., 2016; Melo et al., 2016; Plaza-Diaz et al., 2019). Thus, postbiotic compounds can inhibit the growth of several bacterial pathogens by producing antimetabolites. The presence and characterisation of these compounds require future studying such as by using chromatography.

To evaluate if the principal inhibitory effect is due to the presence of acids, the acidic pH of the CFSMs was neutralised, and their antibacterial activity was observed against both the tested *P. aeruginosa* strains. Our results agree with the results of Shaimaa (2019), who analysed the cell-free supernatant of *L. acidophilus* ATCC 4356, by agar well diffusion and showed its high antibacterial potential against most of the isolated *P. aeruginosa* strains; the same was demonstrated by Shokri-Kojori et al. (2018) in their study that analysed different CFSMs of *Lactobacillus* against *P. aeruginosa*. Both these works have demonstrated that inhibitory activity against *P. aeruginosa* strains was not observed after neutralising the acidic pH of the *Lactobacillus* supernatant. This event could be attributed to the production of antibacterial organic acid molecules, such as lactic, acetic, and formic acids, or possibly to bacteriocins that were active only under acidic conditions (Shokri-Kojori et al., 2018). This affirmation is in accordance with the result of Wala’a and Nibras (2013), where bacteriocins from *L. acidophilus* were found to exhibit activity against *Serratia marcescens* at a pH of 4.7, with half of the activity being lost at pH 8, and then the whole activity being lost at other pH values. Another study of *L. casei* CFSM showed anti-*Shigella* activity *in vitro*, which was abolished after neutralisation (Mirnejad et al., 2013). We can conclude that maintaining natural conditions of the CFSM is imporant, at least with regard to antibacterial activity; also, this behaviour is not specific for one specific pathogen, as reported by many researchers.

To better understand the achieved results, we investigated the antibacterial potential of CFSMs in the growth kinetics of both *P. aeruginosa* strains and observed that a lower percentage of CFSM was used for *L. acidophilus* and *L. delbrueckii* CIDCA 133 (18 or 22% and 20 or 22%, respectively) and a higher percentage for *L. plantarum* and *L. johnsonii* (46 or 48% and 50 or 54%, respectively)*.* At these obtained percentages, the complete inhibition of growth of either PA 9027™ or PA 27853™ was achieved. These results follow the antimicrobial activity, where we observed the best effect (higher inhibition zones) with the CFSM of *L. acidophilus* and in decreasing effectivity with the CFSM of *L. delbrueckii* CIDCA 133, *L. plantarum* and, lastly, *L. johnsonii.* Finally, we analysed the effect of each CFSM against planktonic cells, expressed as the percentage of growth reduction, after analysing the CFU/mL. Independent of the CFSM and the PA tested, we observed a significant reduction (more than 90%) in PA growth. Together, these experiments enable us to infer the anti-pseudomonas activity of all these CFSMs.

Our study has demonstrated that the CFSMs from all tested strains have a strong and moderate inhibition of biofilm formation for all the three tested approaches (co-incubation, pre-coated, and preformed) either to PA 9027™ or PA 27853™.

Many virulent factors are correlated with a pathogen’s initial surface adhesion following the production of extracellular polymeric substances that lead to biofilm formation; this is a phenomenon of microorganisms that results in a persistent microbial mass resistant to antimicrobial agents and is related to approximately 80% of human bacterial infections (Limoli et al., 2015). In biofilms, the microbial population is attached to the surface and maintained by the liberation of an adhesive and protective matrix that provides tolerance and resistance to antimicrobials, which has important implications in healthcare (De Vincenti et al., 2018), especially in burn infections by delaying the healing process. An agent is considered antibacterial if it can achieve a minimum of 3-log/99.9% reduction in bacterial growth (French et al., 2006; Levison and Levison, 2009; Balouiri et al., 2016). Based on our results for planktonic cells, it is possible to infer that the CFSMs of *L. acidophilus* and *L. delbrueckii* significantly reduced the metabolic activity of PA 27853™, with an average measured 4.5-log_10_ reduction. These results follow the antibacterial results obtained in the present study, showing the powerful effect of these CFSM against PA 27853™. The same was not observed for PA 9027™ since all CFSMs from the investigated Lactobacillaceae species demonstrated a reduction in the metabolic activity of *P. aeruginosa*, but only by 2-log_10_. These results are partially in accordance with the study by Jeyanathan et al. (2021) on whole cells of *L. acidophilus*, *L. plantarum*, and *L. reuteri* that showed a minimal impact on PA growth; however, the CFSMs of *L. reuteri* and *L. plantarum* did reveal a reduction in PA growth. Together, this shows the importance of testing each CFSM against each pathogen's microorganism since the effect of the postbiotics is strain dependent, as described for the probiotics.

The CFSMs of different LAB could exhibit other biological activities. Proteins and released exopolysaccharides (EPSs) are some of the biologically active compounds in CFSM (Wang et al., 2020) The transcriptome analyses of the CFSM of *L. acidophilus* A4 showed that the EPSs present exhibit antibiofilm activity by affecting genes that participate in chemotaxis (*cheY*), in Curli amyloid fibre production, which is the component of the extracellular matrix produced by Enterobacteriaceae during biofilm formation of enterohaemorrhagic *E. coli* O157 (Kim et al., 2009). The *cheY* gene is also associated with *P. aeruginosa* biofilm (Barken et al., 2008). The capacity of EPS to inhibit the initial autoaggregation and cell attachment of bacterial cells has also been reported to weaken cell surface modifications or reduce cell-to-cell surface interactions (Kanmani et al., 2013). Based on these reports, we quantified the difference in the total biomass of the biofilms and the metabolic activity present in the formed biomass since *P. aeruginosa* strains are biofilm producers, as shown in our work (positive control, which showed the highest biofilm formation) and according to other reports (Jabalameli et al., 2012; Shokri-Kojori et al., 2018). The biofilm formed by *P. aeruginosa* in the presence of CFSM exhibited a significant reduction in biomass when compared to the control group for PA 9027™ and PA 27853™. The CFSMs for all analysed Lactobacillaceae showed anti-adhesion activity against PA, which could be observed in both the co-incubation and pre-coated approaches, with the metabolic activity at approximately 50%. In the preformed approach, the anti-adhesion activity was not remarkable, and the cell viability was extremely low (less than 20%), except for *L. johnsonii*, which showed cell viability of approximately 40%. Considering the behaviour of CFSMs against PA 9027™, our results show the highest biofilm inhibition percentage of all approaches tested for all CFSMs (between 70% and 80%).

Regarding the percentage of cell viability, our results show values lower than 50%, being statistically different from the positive control. In the preformed approach, it was possible to observe the most insufficient metabolic activity for all tested CFSMs. For the CFSM of *L. acidophilus*, our biofilm inhibition results are in accordance with Shaimaa (2019), who studied the antibacterial and anti-biofilm effect of the CFSM of *L. acidophilus* ATCC 4356 against several *P. aeruginosa* strains, showing that these postbiotics significantly inhibited 68.52% of biofilm formation and removed already preformed biofilms with 43.8% activity. SEM verified the reduction in mass biofilm formation by the CFSM.

Thus, the results of the present work, together with other recent reports in the literature regarding the effect of these probiotics against different pathogens, specifically against *P. aeruginosa*, open new perspectives for biological control agents and could be an exciting approach to replace antibiotics or complement their reduced usage, making these good candidates to overcome the growing challenges of hospital-acquired infections due to this pathogen and other pathogenic microorganisms.

Further studies should address the isolation and characterisation of the bioactive molecules present in the CFSMs studied and the action mechanisms of these postbiotic elements on specific target cells.

## Data Availability

The raw data supporting the conclusion of this article will be made available by the authors, without undue reservation.
